# Risk-sensitive response of soaring birds to crosswind over dangerous sea highlights age-specific differences in migratory performance

**DOI:** 10.1098/rspb.2024.0454

**Published:** 2024-05-29

**Authors:** Carlos D. Santos, Nir Sapir, Paolo Becciu, José P. Granadeiro, Martin Wikelski

**Affiliations:** ^1^ MARE - Marine and Environmental Science Centre and ARNET - Aquatic Research Network Associate Laboratory, Department of Environmental Sciences and Engineering, NOVA School of Science and Technology, NOVA University Lisbon, Caparica 2829-516, Portugal; ^2^ Department of Migration, Max Planck Institute of Animal Behavior, Radolfzell 78315, Germany; ^3^ Animal Flight Laboratory, Department of Evolutionary and Environmental Biology and Institute of Evolution, University of Haifa, Haifa 3498838, Israel; ^4^ Department of Ecology and Evolution, University of Lausanne, Lausanne 1015-CH, Switzerland; ^5^ CESAM - Centro de Estudos do Ambiente e do Mar, Departamento de Biologia Animal, Faculdade de Ciências, Universidade de Lisboa, Lisboa 1749-016, Portugal; ^6^ Department of Biology, University of Konstanz, Konstanz 78457, Germany

**Keywords:** animal tracking, bird migration, ecological barriers, optimal flight, soaring flight, wind drift

## Abstract

Challenges imposed by geographical barriers during migration are selective agents for animals. Juvenile soaring landbirds often cross large water bodies along their migratory path, where they lack updraft support and are vulnerable to harsh weather. However, the consequences of inexperience in accomplishing these water crossings remain largely unquantified. To address this knowledge gap, we tracked the movements of juvenile and adult black kites *Milvus migrans* over the Strait of Gibraltar using high-frequency tracking devices in variable crosswind conditions. We found that juveniles crossed under higher crosswind speeds and at wider sections of the strait compared with adults during easterly winds, which represent a high risk owing to their high speed and steady direction towards the Atlantic Ocean. Juveniles also drifted extensively with easterly winds, contrasting with adults who strongly compensated for lateral displacement through flapping. Age differences were inconspicuous during winds with a west crosswind speed component, as well as for airspeed modulation in all wind conditions. We suggest that the suboptimal sea-crossing behaviour of juvenile black kites may impact their survival rates, either by increasing chances of drowning owing to exhaustion or by depleting critical energy reserves needed to accomplish their first migration.

## 1. Introduction

Bird migration is among the most spectacular phenomena in nature, often involving endurance flights of thousands of kilometres and astonishing concentrations of animals [1]. Migration represents an extreme challenge for most bird species. Many bird migrants cross wide inhospitable areas with reduced opportunities to rest, feed or drink, pushing individuals to the limits of their physiological tolerance and elevating mortality rates significantly [2]. A large proportion of autumn migrants are juveniles that are migrating for the first time. For those who do not follow their elders, orientation is an additional challenge because they lack prior knowledge of migratory routes. Instead, it is generally assumed that they rely on innate compass orientation [3]. Consequently, they are highly susceptible to being blown off track by crosswinds and often undertake suboptimal crossings of ecological barriers [4,5].

For soaring landbirds, large water bodies represent major obstacles during migration because convective updrafts that sustain their flight are typically weak or absent over water [6]. Hence, these birds often undertake remarkable detours from their migratory directions in order to cross water bodies at narrow passages—particularly the most experienced individuals [4,7,8].

For western European populations of soaring birds wintering in Africa, the Strait of Gibraltar is the narrowest crossing of the Mediterranean Sea. Not surprisingly, several species of soaring birds congregate in large numbers in this area during the autumn migration [9]. However, the mere 14 km extent of this sea passage may be illusory with respect to the true challenge it presents to soaring birds. From July to September, when most migratory movement takes place, this area frequently experiences high-speed easterly winds (Levanter winds), which have been demonstrated to severely restrict the passage of soaring birds to Africa [10–12]. Birds attempting to cross in such conditions are forced to engage in prolonged powered flight at very low altitudes over the sea [11–13], which increases the chances of drowning owing to exhaustion. In fact, observations of soaring birds falling into the water while trying to cross the Strait of Gibraltar are relatively frequent [14].

For juvenile black kites (*Milvus migrans*) migrating across the Strait of Gibraltar for the first time, the close proximity of the Moroccan coast, which is clearly visible from the Spanish coast on most summer days, may be a strong incentive to continue migration. Being naive, one may presume that they underestimate the effects of crosswinds and misadventure in risky sea crossings. Thus, as a general prediction in this study, we expect sea-crossing decisions of juvenile back kites to be poorer than those of adult conspecifics. A prior study that showed juveniles perform longer crossings and reach lower heights above the sea than adults does support this assumption [11]. However, some key questions remain unanswered, particularly on how juvenile and adult birds differ on sea-crossing initiation decisions (barrier negotiation), and their wind drift compensation and airspeed modulation on the course of sea crossings. To answer these questions, we combined high-frequency GPS data and triaxial acceleration data recorded from 30 juvenile and 32 adult black kites while crossing the Strait of Gibraltar during the autumn migration with wind data recorded at local weather stations with 10 min resolution. We tested the following specific predictions:

(1) Juveniles are less selective than adults on the crosswind conditions in which to initiate sea crossings. This prediction was based on the results of Sergio *et al*. [15], showing that experienced black kites leave stopovers with more favourable wind conditions than less experienced birds do.

(2) Adults optimize the choice of departure location to minimize the over-water distance to a larger extent than juveniles, possibly anticipating wind displacement during the sea crossing. This prediction relies on the idea that adults use previous experience to optimize sea crossings and are more aware of the risks of suboptimal decisions [3]. The wind displacement anticipation hypothesis has been tested previously for adult white storks (*Ciconia ciconia*) but it was not validated [13].

(3) Adults compensate for wind drift during sea crossings better than juveniles. As in the previous prediction, we assume that adults are more aware of the risks of drifting with crosswinds than juveniles and will be more motivated to control this effect, even though this will elevate the energetic costs of flight. Drift compensation during ecological barrier crossings was demonstrated for adults of two raptor species, ospreys (*Pandion haliaetus*) and marsh harriers (*Circus aeruginosus*) [16]. In addition, drift compensation during migration has been shown to improve with age for black kites [15] and other raptors [5].

(4) Adult and juvenile birds adapt their flapping behaviour to their sea-crossing strategy. Hence, adults are expected to compensate for lateral displacement through flapping, while for juveniles, we expect no relationship between flapping behaviour and lateral displacement if prediction (3) holds. In fact, juvenile black kites were shown to flap less often than adults while crossing the Strait of Gibraltar [11].

(5) Juveniles perform worse than adults in modulating airspeed during sea crossings. Optimal flight theory postulates that birds should increase airspeed with increasing crosswind speed in order to minimize sideways displacement [17] and decrease airspeed with increasing tailwind speed in order to reduce their cost of transport [18].

(6) Adult birds react more to crosswinds blowing towards the Atlantic Ocean than those blowing towards the Mediterranean Sea, while juveniles are expected to behave similarly with crosswinds from both directions. Drifting towards the Atlantic poses higher risks of being dragged to the open ocean than drifting towards the Mediterranean owing to the high speed and steady direction of easterly winds. Adult birds are expected to discriminate better between these two levels of risk than juveniles owing to their earlier experience on this sea crossing. Adult raptors migrating near coasts have been shown to compensate strongly for crosswinds directed towards the ocean [16].

## 2. Methods

### (a) Data collection

**Figure 1.**
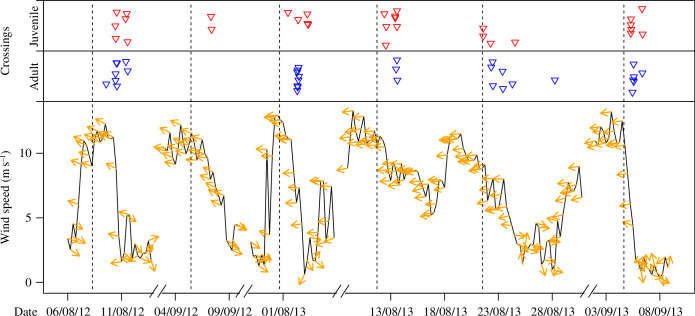
Sea crossings of juvenile and adult black kites relative to changes in wind conditions. Lines represent wind speed and arrows represent wind direction (twice a day). The timeline represents six periods when captures and logger deployments were undertaken. Dashed vertical lines correspond to releases of the tagged birds in each period. Wind data were recorded at weather stations located in Tarifa and Ceuta. Triangles representing bird crossings were jittered vertically to improve readability.

We collected GPS and triaxial acceleration data from 62 black kites (30 juveniles and 32 adults) while they crossed the Strait of Gibraltar during the autumn migration (July to September) in 2012 and 2013. Birds were captured 3.5 km north of Tarifa (36.0426°N, 5.6150°W) in a walk-in trap (7 × 7 × 3.5 m) baited with carrion during periods of high-speed crosswinds (9–13 ms^–1^, figure 1), which are known to restrict sea-crossings to Africa [10–12]. In each capture, we tagged similar numbers of adult birds (second calendar year or older) and juveniles, allowing for inference on age differences in sea-crossing behaviour in similar environmental conditions. Juveniles and adults were distinguished based on moult following Forsman [19]. Juveniles had fresh plumage, while all adults were moulting at the time of capture. Birds were tagged with GPS–GSM data loggers (42 g, TM-202/R9C5 module; Movetech Telemetry, UK) attached as backpacks using Teflon ribbon harnesses. Data loggers were set with baseline schedules to record GPS fixes every minute and a geofence bordering the Strait of Gibraltar that triggered the data recording at a higher frequency. Within the geofence, GPS fixes were recorded every 10 s, with an additional 20 s burst of 1 Hz GPS and three-axial acceleration every 3 min. Recorded data were transmitted via GPRS every 2 h. Further details on field procedures and tracking equipment can be found in Santos *et al*. [20].

### (b) Data analysis

Tracking data (GPS and acceleration) were reduced to the sections overlapping with the Strait of Gibraltar. We further excluded data recorded at less than 500 metres from the coastline of Spain and Morocco to prevent possible soaring behaviour owing to coastal uplift from affecting our results and interpretation. The resulting dataset was then annotated with wind data (speed and direction) of 10 min resolution obtained from local weather stations. Most weather data were recorded at a station in Tarifa (36.0138°N, 5.5988°W), but for two short periods (10–12 August 2012 and 6 September 2013) we used data from a station in Ceuta (35.8886°N, 5.3469°W) owing to the malfunctioning of the Tarifa station. Both stations belong to the Spanish State Meteorological Agency (AEMET).

Testing the predictions (3)–(6) required the use of repeated measurements of flight parameters recorded for each individual bird. Consequently, these data were analyzed using Generalized Estimating Equations (GEE) [21]. We also subsampled the GPS dataset to a fix every 3 min to reduce the temporal autocorrelation in the generated flight and wind parameters. Details on those parameters are presented in electronic supplementary material, table S1 and figure S1. Crosswind and tailwind were calculated for each GPS location as the lateral and forward speed vector components relative to a bird’s target direction, in prediction (3), or a bird’s travel direction, in prediction (5) (electronic supplementary material, figure S1). The nearest distance from each GPS position to the coast of Morrocco (not considering offshore islands) was calculated with the gDistance function of the R package rgeos [22]. We assumed that each bird targeted the closest point at the Moroccan coast at each step of its crossing (electronic supplementary material, figure S2 left panel). Although this assumption may be debatable [23], evidence from birds crossing the Strait of Gibraltar with weak wind showed that they fly along the shortest route to the Moroccan coast (electronic supplementary material, figure S2 right panel). Drift was inferred from birds’ sideways speed component relative to the target direction when subjected to crosswind (electronic supplementary material, figure S1*c*; [5,24]). Full drift assumes that the slope of the linear relationship between crosswind speed and birds’ sideways speed is equal to 1, while full compensation assumes that crosswind speed has no significant effect on birds’ sideways speed [5,24]. Flapping behaviour was inferred from heave amplitude recorded by the accelerometers [25]. Specifically, we classified each acceleration burst as ‘flapping’ if the mean amplitude of the burst readings surpassed a threshold established from video recordings of tagged birds during takeoff or ‘non-flapping’ otherwise (electronic supplementary material, figure S1*d*).

Age-related differences were examined separately for birds crossing during winds with positive and negative east–west speed components, hereafter referred to as ‘east component winds’ and ‘west component winds’, respectively. These two wind conditions are usually segregated in time by several days (see figure 1) and are known to influence sea-crossing behaviour of soaring birds [10–12]. East component winds (also called Levanter winds) are particularly strong and have a steady direction (figure 1; [26]). In contrast, west component winds are usually weaker and have heterogeneous directions, posing less risk for the birds (figure 1). For this reason, we expected the birds to behave differently to cope with the different risks presented by the two wind conditions. For flapping behaviour, we only drew comparisons for east component winds owing to the reduced amount of acceleration bursts recorded for adults crossing with west component winds.

All GEE models were fitted with bird identity as a cluster identifier and assumed an independent correlation structure for the data within clusters. This was supported by the reduced temporal autocorrelation of model residuals (electronic supplementary material, figure S3). The flapping probability model was fitted with a binomial distribution owing to the dichotomous nature of the dependent variable (‘flapping’ versus ‘non-flapping’). All of the remaining GEE models assumed Gaussian distributions.

GEE models were built in R using the function geeglm of the geepack package [27]. Temporal autocorrelation of model residuals was computed with the function acf of the stats package [28].

## 3. Results

Most black kites timed the sea crossing day with wind speed drops (44 out of 62; figure 1), which typically occurred during the night after periods of several days of strong easterly wind. There was no significant age difference in the proportion of birds crossing in the days of wind drop and other days (crossings in wind drop days: 26 out of 32 for adults and 18 out of 30 in juveniles; chi-square test: *χ* ^2^ = 2.44, *p*‐value = 0.12; figure 1). Seven birds (six of which were juveniles) crossed during days before wind speed drops, experiencing high-speed easterly winds (exceeding 10 ms^–1^) during their sea crossings.

**Figure 2.**
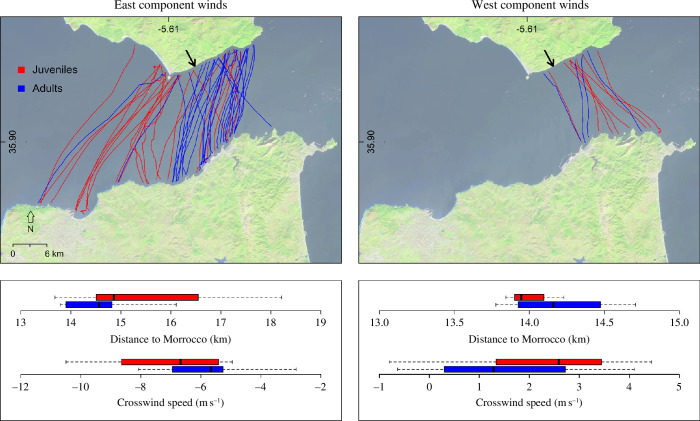
Sea crossings of juvenile and adult black kites during winds with an east or a west speed component. Black arrows in the top panels indicate the shortest distance to Morocco. Boxplots show age differences in the distance to Morocco from the crossing starting point and in the crosswind speed experienced by the birds during the sea crossing. Crosswind speed component calculation assumed that the birds targeted the closest point at the Moroccan coast at each step of their crossing.

The vast majority of birds (50 out of 62) crossed during winds with an east speed component (figure 2). In those wind conditions, juveniles crossed with crosswinds of higher speed than adults (*t*‐test: *t* = 2.23, *p*‐value = 0.03; figure 2). In addition, they initiated their crossings more distantly from the Moroccan coast than adults (*t*‐test: *t* = 2.62, *p*‐value = 0.015; figure 2). It should be emphasized that most adults initiated their crossings eastwards from the narrowest point to the Moroccan coast, while no similar pattern was evident in juveniles (figure 2). During winds with a west speed component, sea crossings of adults and juveniles were performed under similar crosswind speeds and started from locations at similar distance from the Moroccan coast (*t*‐test crosswind speed: *t* = 0.69, *p*‐value = 0.518; *t*‐test distance to Morocco: *t* = 0.76, *p*‐value = 0.486; figure 2).

**Figure 3.**
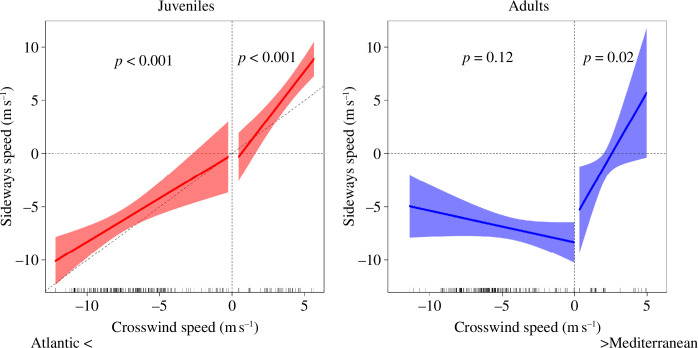
Drift of juvenile and adult black kites during sea crossings. Plots show partial effects of crosswind speed on the birds’ sideways speed based on four different GEE models (one for each age class in each wind condition, i.e. winds with east or west speed component). See electronic supplementary material, table S1 for model details. Crosswind and sideways speed component calculations assumed that birds targeted the closest point at the Moroccan coast at each step of their crossing. Shading represents 95% confidence intervals. Dashed oblique line in the left panel represents full drift, i.e. when birds deviate from the target in a direct proportion to crosswind speed.

Adults compensated for wind drift under easterly winds, showing no significant trend in sideways speed with the increase of crosswind speed component after correcting for the tailwind speed component (figure 3; electronic supplementary material, table S1). However, adults drifted under winds with a west speed component, showing an increasing trend of sideways speed with an increasing crosswind speed component (figure 3; electronic supplementary material, table S1). Juveniles drifted under winds with east and west speed components, showing significant relationships between sideways speed and the crosswind speed component (figure 3; electronic supplementary material, table S1).

**Figure 4.**
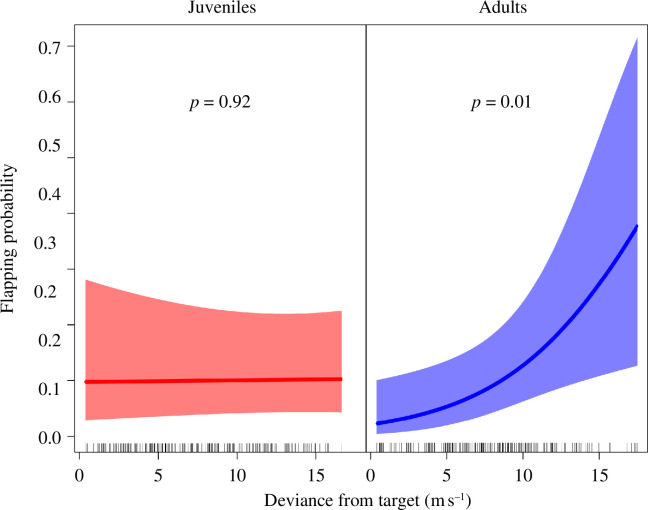
Flapping behaviour of juvenile and adult black kites relative to their deviance from the closest point in the Moroccan coast (target) during sea crossings performed under winds with an east speed component. ‘Deviance from the target’ is absolute sideways speed considering the closest point on the Moroccan coast as the sea crossing goal. Flapping behaviour derived from 1 Hz acceleration bursts recorded for 20 s every 3 min during the sea crossings. Bursts were converted into flapping or non-flapping based on a heave amplitude threshold established from video recordings of tagged birds during takeoff (see electronic supplementary material, figure S1). Model details are shown in electronic supplementary material, table S1. Shading represents 95% confidence intervals.

**Figure 5.**
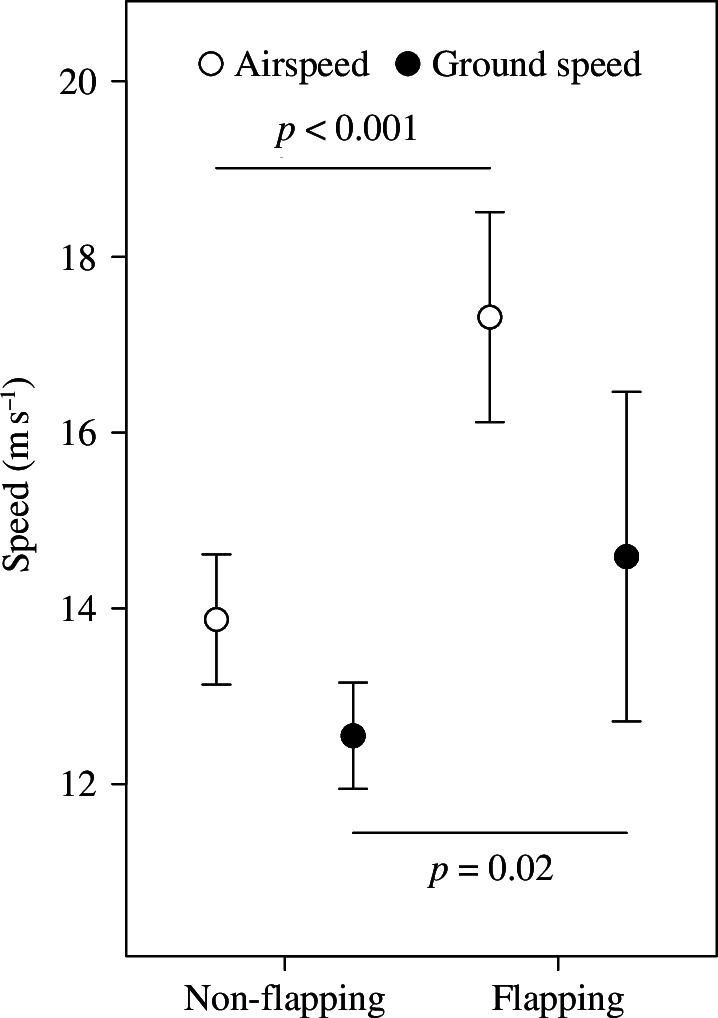
Effects of flapping of adult black kites on their airspeed and ground speed in sea crossings during winds with an east speed component. Flapping behaviour derived from 1 Hz acceleration bursts recorded for 20 s each 3 min during the sea crossings. Bursts were converted into flapping or non-flapping based on a heave amplitude threshold established from video recordings of tagged birds during takeoff (see electronic supplementary material, figure S1). Model details are shown in electronic supplementary material, table S1. Error bars represent 95% confidence intervals.

During sea crossings with easterly winds, adult black kites significantly increased their probability of flapping with increasing lateral displacement, while juveniles showed no significant trend in this relationship (figure 4; electronic supplementary material, table S1). Flapping behaviour of adults in these wind conditions resulted in significant increases in airspeed and ground speed (figure 5; electronic supplementary material, table S1).

**Figure 6.**
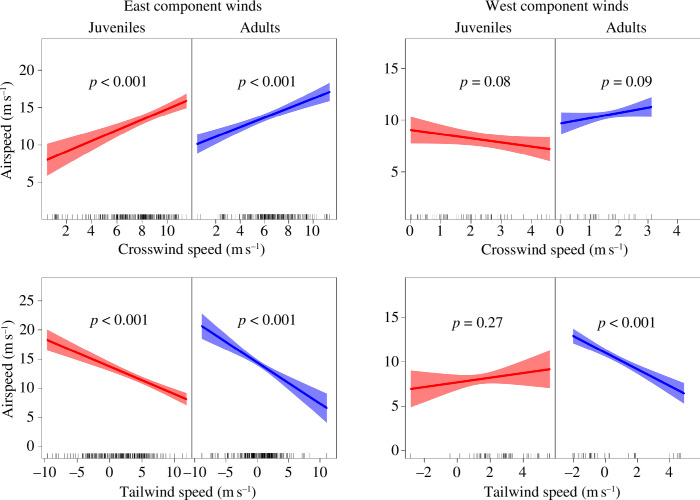
Effects of tailwind and crosswind speed on airspeed for juvenile and adult black kites crossing the Strait of Gibraltar during winds with an east or a west speed component. Crosswind and tailwind speed components were calculated regarding the travel direction of each bird in each GPS fix. Crosswind speed component was expressed in absolute values (no distinction of blowing from left or right). The plots represent partial effects of GEE models that simultaneously account for tailwind and crosswind speeds as predictors (see electronic supplementary material, table S1 for model details). Shading represents 95% confidence intervals.

**Figure 7.**
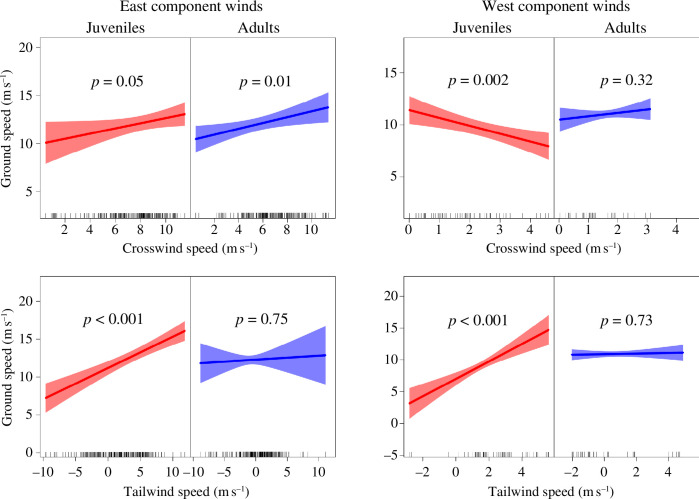
Effects of tailwind and crosswind speed on ground speed for juvenile and adult black kites crossing the Strait of Gibraltar during winds with an east or a west speed component. Crosswind and tailwind speed components were calculated regarding the travel direction of each bird in each GPS fix. Crosswind speed component was expressed in absolute values (no distinction of blowing from left or right). The plots represent partial effects of GEE models that simultaneously account for tailwind and crosswind speeds as predictors (see electronic supplementary material, table S1 for model details). Shading represents 95% confidence intervals.

Adults and juveniles significantly increased airspeed and ground speed with increasing crosswind speed component under easterly winds (figures 6 and 7; electronic supplementary material, table S1). No significant trend of that kind was observed under winds with a west speed component, although there was a significant decline in ground speed with increasing crosswind speed component for juveniles (figures 6 and 7; electronic supplementary material, table S1). Both age classes also responded to increasing tailwind speed component by significantly decreasing airspeed during easterly winds (figure 6; electronic supplementary material, table S1). This resulted in a non-significant effect of tailwind speed component on ground speed of adults, while for juveniles there was still a significant positive effect of tailwind speed component on ground speed (figure 7; electronic supplementary material, table S1). During winds with a west speed component, the patterns described above were similar for adults, while juveniles showed no significant effect of tailwind speed component on airspeed but repeated the increasing trend of tailwind speed component on ground speed (figures 6 and 7; electronic supplementary material, table S1).

## 4. Discussion

Juvenile black kites followed our prediction (1) for barrier negotiation only partially. Hence, the majority of tracked birds timed their sea crossings to coincide with decreases in wind speed following periods of several days of strong easterly crosswinds, mirroring the behaviour of adult birds (figure 1). This may suggest that juveniles relied on social information to choose the day of crossing, avoiding crossing on days when adult conspecifics or even other soaring bird species did not attempt to cross. However, juveniles tended to cross with higher easterly crosswind speed compared with adults (figure 2), suggesting that, despite their optimal choice of the crossing day, they were unable to fine-tune the crossing departure time as efficiently as adults. Furthermore, they were less selective than adults in the choice of the departure location to minimize the crossing distance during easterly winds (figure 2), in line with our prediction (2). Interestingly, the vast majority of adults chose departure locations eastwards from the narrowest point to the Moroccan coast during easterly winds (figure 2), supporting the idea that they anticipated westwards drift during the sea crossing. The lack of age-related differences during winds with a west speed component may indicate that adults did not try to minimize crosswind speed or crossing distance owing to the low risk posed by such wind conditions. However, we cannot exclude that this result is related to the low number of tracks recorded, lowering the power of statistical tests (figure 2).

Once crossing was initiated, adults compensated for winds with an east speed component but drifted under winds with a west speed component (figure 3). This contrasting behaviour seems to indicate that adult birds perceive easterly crosswinds as a higher risk for their survival owing to their high speed and steady direction (figure 1), having the potential to drag them out over the open Atlantic Ocean or to force long crossings dependent on powered flight [11–13]. One should note, however, that sideways speed was ubiquitously lower than crosswind speed (i.e. the regression intercepts below zero; figure 3), meaning that birds ended up westwards of the closest point on the Moroccan coast relative to the departure location (figure 2). This evidence, as well as the observed drift towards the Mediterranean Sea, may be interpreted as adaptive drift where birds tolerate a certain level of displacement that does not represent a high risk of ending up at open sea and may be compensated for later in the migration [29,30]. This strategy may be translated into important energy savings that otherwise would be wasted in powered flight [29,30]. The negative intercept of the regression for wind with a west speed component (figure 3) was owing to a cluster of observations at low wind speed (electronic supplementary material, figure S4) derived from two individuals that did not try to minimize crossing distance (figure 2), possibly because the benign wind conditions did not challenge their sea crossing.

In sharp contrast with the behaviour of adult birds, juveniles tended to drift almost fully under winds with both east and west speed components (figure 3). This suggests that juveniles underestimated the high risk of easterly crosswinds, as expected by their lack of experience in crossing the Strait of Gibraltar. However, we cannot exclude that juveniles may have been simply unable to compensate for crosswind over water owing to inferior flight performance, which has been shown in other soaring birds [31,32]. Taken together, these results support our prediction (3) that adults are more willing to avoid wind drift than juveniles and (6) that they recognize the higher risk of winds blowing towards the Atlantic Ocean compared with those blowing towards the Mediterranean Sea.

We were also able to demonstrate that adults corrected for lateral displacement during easterly wind through flapping. Hence, acceleration data indicated that the probability of flapping increased with sideways speed, resulting in higher airspeed and ground speed (figures 4 and 5). In contrast, juveniles showed no significant change in flapping probability with increasing lateral displacement (figure 4). This supports the idea that they did not try to counteract wind drift. Overall, these age differences in flapping behaviour are in accordance with our prediction (4).

Contrary to our expectations in prediction (5), juvenile and adult birds seemed to modulate airspeed similarly in response to crosswind speed. However, juveniles showed a poorer use of tailwind to reduce the cost of transport. Overall, our results match optimal flight theoretical predictions to a larger extent for winds with an eastern speed component than those with a west speed component.

Both age classes increased airspeed for increasing easterly crosswind speed (figure 6), which is in accordance with theoretical predictions for optimal flight [17] and empirical results from other bird species and bats [13,24,33,34]. However, this similar pattern may have resulted from two different sea-crossing strategies. While adults seem to have increased airspeed to actively compensate for drift, as shown in the results reported above (figures 3–5), the reduced drift compensation of juveniles suggests that they tried to overcome crosswind over sea by travelling as quickly as possible, independently of their trajectory. By allowing for lateral displacement, juveniles made more use of the tailwind speed component, thus allowing them to travel faster.

We also found similar patterns between the two age classes in airspeed modulation relative to tailwind speed during easterly wind. Both juveniles and adults reduced their airspeed with increasing tailwind speed (figure 6), which helps them to minimize their cost of transport and sink rate [18]. Similar findings have been reported for several other soaring birds [13,33,35,36]. We should note, however, that while adults modulated airspeed to the extent that they kept their ground speed constant, juveniles showed an increase in ground speed with increasing tailwind speed (figures 6 and 7). This suggests that adults have a superior capacity to use tailwinds.

Airspeed modulation was less obvious as a response to winds with a west speed component, possibly owing to the reduced range of crosswind and tailwind speeds. Neither juveniles nor adults changed their airspeed significantly with increasing crosswind speed, but, strangely, ground speed decreased with increasing crosswind speed for juveniles (figures 6 and 7). This could be a spurious result owing to the low number of observations used in the linear regression model. Airspeed responses of adults to tailwind speed were similar to those observed for easterly wind, reducing the cost of transport by decreasing airspeed with increasing tailwind speed to the extent that no significant change was observed in ground speed (figures 6 and 7). By contrast, juveniles showed no change in airspeed with increasing tailwind speed, and consequently, their ground speed significantly increased (figures 6 and 7).

Overall, our results show highly contrasting sea-crossing behaviours between juvenile and adult black kites and they match our general prediction that adults make optimal decisions based on their previous experience, while juveniles underestimate the challenge and potential risks of crossing the Strait of Gibraltar because they lack previous knowledge to rely on. Optimizing decisions is critical for soaring bird survival during sea crossings. There are records of soaring birds falling into the water during strong crosswinds [37] or owing to route misjudgment [4,38]. Black kites often abort the crossing of the Strait of Gibraltar, sometimes landing onshore before resuming migration, presumably as a result of exhaustion [11]. Juveniles, in particular, undertake exceptionally long crossings and fly at dangerously low altitudes above the sea [11]. Here, we show that they also naively disregard easterly crosswind drift compensation.

Although the increased mortality of soaring birds during their first migration is widely reported in the literature [4,32,39,40] and considered a natural trait of population dynamics, the increasing frequency of harsh weather owing to climate change may tilt differences between adult and juvenile survival even further, with important population consequences. We should emphasize that this potential threat adds up to a multiplicity of human-related factors that are causing severe declines in many populations of soaring birds.

## Data Availability

The data used for this study are available through the Movebank Data Repository [41]. Supplementary material is available online [42].
